# Recent Progress in the Synergistic Bactericidal Effect of High Pressure and Temperature Processing in Fruits and Vegetables and Related Kinetics

**DOI:** 10.3390/foods11223698

**Published:** 2022-11-18

**Authors:** Sinan Zhang, Maninder Meenu, Lihui Hu, Junde Ren, Hosahalli S. Ramaswamy, Yong Yu

**Affiliations:** 1 College of Biosystems Engineering and Food Science, Zhejiang University, 866 Yuhangtang Road, Hangzhou 310058, China; 2 Key Laboratory of Equipment and Informatization in Environment Controlled Agriculture, Ministry of Agriculture, 866 Yuhangtang Road, Hangzhou 310058, China; 3 Hangzhou Jiangnan Talent Service Co., Ltd., 681 Qingchun East Road, Hangzhou 310000, China; 4 Department of Food Science and Agricultural Chemistry, McGill University, 21111 Lakeshore Road, St-Anne-de-Bellevue, QC H9X 3V9, Canada

**Keywords:** fruit and vegetable products, high-pressure, high-pressure thermal processing, low temperature, bactericidal kinetics model

## Abstract

Background: Traditional thermal processing is a widely used method to ensure food safety. However, thermal processing leads to a significant decline in food quality, especially in the case of fruits and vegetables. To overcome this drawback, researchers are extensively exploring alternative non-thermal High-Pressure Processing (HPP) technology to ensure microbial safety and retaining the sensory and nutritional quality of food. However, HPP is unable to inactivate the spores of some pathogenic bacteria; thus, HPP in conjunction with moderate- and low-temperature is employed for inactivating the spores of harmful microorganisms. Scope and approach: In this paper, the inactivation effect of high-pressure and high-pressure thermal processing (HPTP) on harmful microorganisms in different food systems, along with the bactericidal kinetics model followed by HPP in certain food samples, have been reviewed. In addition, the effects of different factors such as microorganism species and growth stage, process parameters and pressurization mode, and food composition on microbial inactivation under the combined high-pressure and moderate/low-temperature treatment were discussed. Key findings and conclusions: The establishment of a reliable bactericidal kinetic model and accurate prediction of microbial inactivation will be helpful for industrial design, development, and optimization of safe HPP and HPTP treatment conditions.

## 1. Introduction

Food safety has become a global common concern as consumers around the world are facing different degrees of food safety risks. As per the reports of the World Health Organization (WHO), approximately 600 million people (almost 1 in 10 people in the world) fall ill after consuming contaminated food, and approximately 420,000 die every year due to foodborne diseases [1]. Most foodborne diseases are more likely to be widespread and, even global due to changes in food production, supply, and widespread food distribution. New foodborne pathogens continue to be found as a result of altered food production conditions and improved laboratory detection methods. In particular, a significant increase in antimicrobial drug-resistant bacteria and several viruses was observed, which were not previously recognized [2]. The food industry all over the world is also facing huge economic losses due to the contamination of raw materials. Therefore, food safety remains a huge global challenge as foodborne diseases obstruct socioeconomic progress by straining healthcare systems and harming national economies, international trade, and tourism. Therefore, sterilization has become the utmost important step in food processing industries. It was also observed that the detection of food pathogens, food contaminants, and toxins has also received significant attention since the last decade to ensure food safety [3].

Conventionally, several chemical and physical methods have been used for the decontamination of food material. However, these methods are associated with several drawbacks such as chemical residue in resultant food products and poor sensory and nutritional quality of food. Thus, these conventional decontamination methods were not able to meet consumers’ increasing demand for high-quality food. At present, thermal sterilization is the mainstream sterilization method in the food processing industry. Although thermal processing ensures product safety, it causes undesirable nutritional and organoleptic quality in food [4,5,6]. *Alicyclobacillus acidoterrestris* is a type of bacillus that can grow in pasteurized beverages. It has been mentioned that the use of high temperatures during heat treatment can inactivate *A. acidoterrestris* inoculated in apple juice, but also deteriorate the nutritional and sensory qualities of apple juice [7]. Among various food products, fruit and vegetable products, the majority of which contain heat-sensitive ingredients, exhibit significant degradation in their nutritional value, appearance, taste, and flavor following heat treatment [8,9]. Some fruits, such as carambola [10] and muskmelon [11], contain volatile sulfur compounds, which are responsible for their unique flavor. It was also mentioned that degradation of these compounds following heat treatment resulted in off-flavor and the low market price of these food products. That, in turn, limits the commercial production of these fruit and resultant products. In addition, the thermal processing of food materials also resulted in a significant decrease in their antioxidant capacity [10,12,13]. Therefore, it is urgent to adopt an alternative method that can not only enhance the microbial safety of food but also maximize the retention of the original physical and biochemical properties of food.

High-pressure processing (HPP) sterilization is one of the promising techniques among recent technologies that employs low temperature and high pressure, which can kill the vast majority of harmful microorganisms in the food in just a few minutes [14]. HPP is a non-thermal food processing technology that was reported to maintain the original flavor, physical parameters, and chemical properties of heat-sensitive fruit pulp to their maximum extent [15,16]. In comparison with traditional thermal processing, HPP presents better retention levels of the bioactive compounds, increased microbial safety and reduced enzyme activity [10,17]. Thus, HPP technology has been regarded as a green alternative to traditional preservation technologies. In the past 20 years, HPP technology has been industrialized in the field of food processing in some countries and regions to meet consumer demand for mild processed food without preservatives and with high nutritional quality.

HPP is governed mainly by Le Chatelier’s principle, the microscopic order principle, and the Isostatic principle. Le Chatelier’s principle states that any process in equilibrium that is accompanied by a decrease in volume can be enhanced by pressure. A change in micro-order also occurs during HPP treatment, which means that the molecules will shift to a more compact structure. The Isostatic principle states that the applied pressure is instantaneously and evenly distributed within the food, independent of the structure and geometry of the food [18,19]. The mechanisms of microbial inactivation by HPP are based on a combination of changes in the cell membranes. Structural changes in protein and membrane phospholipids can alter membrane permeability and cellular functions [20].

Along with potent advantages, HPP technology also exhibits certain limitations. In general, spoilage bacteria and pathogenic microorganisms are inactivated in the pressure range of 400 to 600 MPa, but certain microorganisms exhibit strong pressure tolerance, especially Gram-positive bacteria. In addition, pressure treatments up to 1000 MPa may not completely inactivate bacterial spores at ambient temperatures [21,22]. It is important to note that the pasteurization of foods requires at least 5–6 log cfu/g or ml reduction of key pathogenic or spoilage microorganisms. Traditional thermal processing of food products can efficiently achieve the required reduction of pathogenic or spoilage microbes. However, it also diminishes the quality of fruits and vegetables [23]. Thus, a combination of HPP and moderate temperature treatment is required for the efficient inactivation of spores while retaining the physical and nutritional quality of food.

Different fruit and vegetable products have different physical and biochemical characteristics, and during decontamination the different parameters of different processing methods have to be optimized to achieve optimum product quality, microbial safety, and other aspects. Therefore, much basic research is required to provide a theoretical basis for the practical application of a particular processing method. Through bactericidal kinetics, the lethal characteristics of microorganisms can be described, which plays an important role in predicting the number of microorganisms and ensuring the microbial safety of fruit and vegetable products [24]. These kinetic models can also be used to develop Hazard Analysis and Critical Control Point (HACCP) plans and process validation studies.

A reliable mathematical model and an accurate prediction of microbial inactivation will be helpful for the industrial design, development, and optimization of safe decontamination treatment, which in turn reduces the number of experiments [25,26]. Therefore, significant efforts are required to obtain different kinetic model parameters for various target bacteria in order to develop a database. Thus, this paper is an initiative to review the research progress related to the bactericidal kinetics of HPP and temperature–pressure synergy in fruit and vegetable products for microbial inactivation and food safety enhancement.

## 2. Inactivation Kinetics of Microbes by Temperature in Conjunction with High-Pressure

### 2.1. Primary Models

Primary models in the bactericidal kinetics of HPTP are mathematical equations that describe the changes in microbial counts induced by pressure as a function of treatment times. The first-order kinetic model is one of the common primary models employed to describe microbial log survivors in foods followed by the combination of high pressure and temperature pressure treatment [23,27]. The first-order dynamic model is explained as follows:

(1) $$Log\left(\frac{N}{N_{0}}\right)=-kt=-\frac{t}{D_{T,P}}$$

where *N*_0_ is the initial number of viable cells in the control sample (cfu/g or cfu/mL), *N* is the number of survivors in samples after HPP or HPTP treatment, *t* is the processing time, *k* is the inactivation rate constant of microbial number (related to the environmental conditions), and *D_T,P_* is the time required for one log reduction of the microbial population at a certain temperature and/or pressure.

The HPP and high-pressure thermal processing (HPTP) showed a linear trend for microbial inactivation in some fruit and vegetable systems, as mentioned in Table 1. However, in some cases, the inactivation curve of microorganisms may present a delayed or tailing phenomenon as the pressure treatment may cause sublethal injuries in the microorganisms and may activate dormant spores [28,29]. The most accepted hypothesis used to explain the tailing effect is “the presence of subpopulations within a microbial population that are more resistant to pressure treatments and remain viable even after prolonged pressure holding times” [30]. In addition, the non-linear behavior during pressure treatments is attributed to the cumulative damage to microbial cells [31]. In this case, the linear model of first-order kinetics can no longer fit the situation, and a nonlinear model is required to explain the delayed or tailing effect. Similar to the first-order kinetic model, the Weibull model is also a common primary model, and it is a typical representation of non-linear models. The Weibull model is not only applicable to describe microbial thermal inactivation [27] but can also be employed to explain other sterilization methods, such as pulsed electric field [32] and HPP [33]. Some studies have also mentioned that the Weibull model was more suitable to describe the microbial inactivation caused by the HPTP compared with thermal inactivation (Table 1). Hossein et al. reported that Weibull distribution was the best mathematical model to describe the inactivation of *Bacillus coagulans* 185A spores in tomato juice by HPTP, whereas first-order kinetics was appropriate for explaining only thermal processing [34].

The Weibull model was initially used for the determination of failure time in reliability engineering and was applied to the survival curve of microorganisms, which was the combination of the accelerated failure time model and parameter distribution [38,39]. The Weibull model (Equation (2)) was based on the principle of heterogeneity in the resistance distributed among individual cells within a population [40].

(2) $$log\frac{N}{N_{0}}=-bt^{n}$$

where *b* is a rate parameter that is related to the rate at which the microorganism is inactivated, and *n* describes the degree of curvilinearity. When *b* < 1, the inactivation curve corresponds to concave-upwards (tailings). When *b* > 1, the inactivation curve corresponds to concave-downwards (shoulders). When *b* = 1, the model becomes a straight line, which is the first-order kinetic model [41]. This model assumes that the probability of death of a single cell or spore after treatment is dispersed according to Weibull distribution, and the survival curve of the distribution of lethal events is exponentially cumulative. The Weibull model also assumes that the microbes exhibit different resistance against treatment and these differences are permanent [42].

### 2.2. Secondary Models

The first-order kinetic models are established under certain temperature and pressure conditions. Unlike first-order kinetic models, the secondary models are an extension of the primary models, in which the parameters of the primary models relate to the environmental variables/conditions such as pressure or temperature [23]. Secondary models are applied to predict changes in the kinetics parameters of primary inactivation models as functions of intrinsic or extrinsic factors [43]. *Z_T_*-value (shown in Equation (3)) represents the temperature required to decrease D by one log cycle under a certain pressure, which can also reflect the sensitivity of microorganisms to temperature. This is equal to the reciprocal of the slope of the log *D*-values plotted against temperature [44,45]. The smaller the *Z_T_*-value, the higher the temperature sensitivity will be.

(3) $$Z_{T}=\frac{T_{1}-T}{logD-logD_{1}}$$

where *D*_1_ is the *D*-value at a reference temperature *T*_1_ (°C) and *T* is the temperature of the isothermal treatment (°C). Likewise, the *Z_P_*-value (shown in Equation (4)) is the pressure required to decrease the *D* value by one log cycle at a certain temperature and is equal to the reciprocal of the slope of the log *D*-values plotted against pressure. *Z_P_*-value can also reflect the sensitivity of microorganisms to pressure. A smaller *Z_P_*-value indicates greater sensitivity to pressure.

(4) $$Z_{P}=\frac{P_{1}-P}{logD-logD_{1}}$$

The previous studies related to high-pressure and temperature synergistic bactericidal kinetics also reflect the importance of sample temperature detection and control technology under a high-pressure environment. If the sample temperature under a high-pressure environment is not accurately detected and efficiently controlled, then the model developed to predict bacterial kinetics will be inaccurate [22,46]. For example, Lori et al. used a first-order kinetic model to accurately describe the thermal and pressure inactivation mechanics of *Campylobacter coli* and *C. jejuni* [47]. At the same time, secondary models were established to accurately predict the changes in the number of *C. coli* and *C. jejuni* under the combined effects of pressure (0.1–500 MPa), temperature (10 to 65 °C), and treatment time. In addition, the Weibull model can also relate the inactivation rate parameter (*b*-value) to environmental variables, particularly temperature, and can be used to predict the parameter values outside the range of the variables tested [23].

### 2.3. Polynomial Models

Polynomial models, also known as Response Surface Methodology (RSM), are used to analyze the effect of individual factors on inactivation parameters or their interactions [23,43]. RSM can provide an optimal fitting of polynomial models from a minimal number of experiments and enable the interaction study between factors based on the response of interest, including nonlinearities on curves [26,43]. As shown in Equation (5), described by Evelyn and Filipa [23]:

(5) $$Y=B_{0}+\overset{n}{\underset{i=1}{\sum }}B_{i}X_{i}+\overset{n}{\underset{i=1}{\sum }}B_{ii}X_{i}^{2}+\overset{n}{\underset{j\neq 1}{\sum }}B_{ij}\;X_{i}X_{j}+\varepsilon$$

where *Y* is the predicted response; *B*_0_ is a constant; *B_i_*, *B_ii_*, and *B_ij_* are model coefficients; *X_i_* and *X_j_* are the input variables (environmental factors); and *ε* is the error term. By graphically translating RSM, operators can find the operating conditions that reduce the response, *Y*.

## 3. Influence of Different Factors on Microbial Inactivation during HPP or HPTP

Many factors affect the inactivation of microorganisms during HPP or HPTP. These factors include microbial species and growth stage, process parameters and pressurization method, temperature under pressure, food composition, and other factors such as food additives, water activity, and pH value [48,49]. in addition, researchers have also explained the influence of process parameters and pressure mode on microbial inactivation, and their mechanism of action has also been widely recognized. The research on high-pressure synergistic sterilization at moderate temperatures is also increasing. However, only a few studies have reported the synergistic sterilization of food products by low temperature and high pressure. It is important to note that treatment temperature exhibits a significant effect on the development of the kinetic model for sterilization [50].

### 3.1. Microbial Species and Growth Stages

Different microbe species have different sensitivities to high pressure, even within the same food system. In addition, microorganisms, including pathogens, can present significantly different responses toward high pressure. This variation exists not only between different species but also between strains of the same species [48]. Table 2 shows the inactivation effect of high pressure on different kinds of microorganisms in the same food systems. The pressure tolerance order of general microorganisms is highest in spores > Gram-positive (*Listeria monocytogenes* and *Staphylococcus aureus*) bacteria > fungus > Gram-negative (*Escherichia coli*) bacteria [48,51,52]. However, this order can be changed based on the food system. This is mainly because some macromolecules in food can play a protective role on some microorganisms. In addition, the resistance of microorganisms to high pressure is varied based on their growth stages. The pressure tolerance of microbial cells in their stable growth phase is higher compared to the cells in the logarithmic phase [53,54,55,56,57,58,59].

Until now, inactivated bacterial spores are the biggest challenge faced by HPP during food sterilization. Bacterial spores are highly resistant compared to mold and yeast spores. These bacterial spores are likely to grow as toxin-producing cells, leading to foodborne illness and disease outbreaks. Therefore, they are often used as indicators of pasteurization and sterilization processes in foods. Conventional pressures (<600 MPa) in the food industry have difficulty inactivating the majority of spores at room temperature. Even under the extreme pressure generated by laboratory equipment, some spores can survive. For example, *B. subtilis* spores were found to survive under processing conditions up to 1200 MPa at ambient temperature [57]. In addition, spore resistance varies significantly between species and is significantly influenced by the food substrate. In general, among all microorganisms, spores exhibit the highest resistance to HPP, thus presenting an urgent need for HPTP for their inactivation in foods [23].

### 3.2. Process Parameters and Pressure Mode

The influence of pressure and holding time of HPP or HPTP on the bactericidal effect has been studied extensively [48]. Generally, a higher pressure and a longer holding time correspond to a better sterilization effect [48,58,59,62]. Margaret et al. [63] studied the effect of HPP (at 20 °C for 1 min) in phosphate buffer on Leuconostoc and found a significant decrease in the number of Leu. kimchii in the phosphate buffer with an increase in the pressure level. Basak et al. [64] also mentioned that under the same level of pressure, the mortality of Leu. mesenteroides in orange juice increased with the increase of pressure hold time. However, the lethality of some microbial spores under low pressure may be higher compared to the mortality under high pressure as higher pressure may associate with spore germination [23,65].

The variation in pressure applied during HPP also exhibits a significant effect on microbial inactivation. The most commonly employed method is a single static high pressure (pressure holding time > 0 s). The whole high-pressure treatment process includes three parts: boost pressure, keep pressure, and relieve pressure. The boost and relief process without the keep pressure process during high-pressure treatment is called pulse-type high-pressure treatment. Different types of high-pressure pasteurization methods are composed of these two above-mentioned methods [66]. It has been reported that multi-stage high pressure (with or without pressure holding time) treatment can improve the microbial inactivation rate [67,68,69]. The multi-pulsed HPP is mentioned to be more effective than classical or single-pulsed HPP for the inactivation of enzymes, yeast cells, bacterial cells, and fungal and bacterial spores [70]. Aleman et al. have also found that multi-pulse HPP treatment was more effective compared to the single-pulse HPP treatment for inactivating *Saccharomyces cerevisiae* in pineapple juice at the same holding time [71]. Donsì et al. also indicated that the effectiveness of multiple pulses is dependent on the combination of pulse number, pressure, and temperature [62].

Chapleau et al. found a linear reduction in microbial count (*Salmonella typhimurium* and *L. monocytogenes*) with high-pressure pulses as a product of pressure and time [72]. Meanwhile, a logarithmic reduction in the microbial count was observed with increasing holding time. In addition, the pressurization and depressurization rates also have an impact on the sterilization effect [72,73]. Furthermore, Ratphitagsanti et al. have also reported the potent bactericidal effect of a low rate of pressurization during HPP compared to the high-rate of pressurization of samples containing *B. amyloliquefaciens* spores [73]. This study also found that double-pulse treatment presents better sterilization compared to single-pulse treatment. 

### 3.3. Temperature

Temperature is the most important external condition for microbial growth and metabolism, and it has a significant influence on microbial survival. It is well known that the ambient temperature around the sample during pressurization will affect microbial resistance. It is also mentioned that moderate and low temperatures will increase the inactivation rate of microorganisms compared to room temperature [48]. However, there are significant differences in the sterilization mechanism of high-pressure and moderate-temperature synergistic sterilization and high-pressure and low-temperature synergistic sterilization. In the following sections, studies related to both sterilization methods are discussed in detail.

#### 3.3.1. High-Pressure and Moderate Temperature Synergistic Sterilization in Fruits and Vegetables

The sterilization effect of high-pressure treatment will be strengthened with the increase in temperature, especially above room temperature (25 °C). It has been mentioned that the treatment at a moderate temperature (40–90 °C) increases the degree of protein denaturation; thus, an increase in the temperature during HPP treatment significantly enhances the lethality of microorganisms [74]. On the other hand, high pressure can lead to irreversible alteration in microbial cellular structure. The high pressure causes damage to both the membrane and the cell wall, which increases cell permeability and leads to an interruption in cell metabolism [49,75]. In addition, the loss of the secondary, tertiary, or quaternary structure of large molecules and the modification of complex organized structures are observed, followed by high-pressure treatment, which, in turn, leads to microbial death [76]. Furthermore, Zhang et al. have also mentioned a decrease in the thermal stability of horseradish peroxidase with an increase in the pressure [14]. High-pressure treatment alters the structure of the protein, which in turn changes the stability of the protein under pressure. In addition, moderate heating can enhance microbial inactivation under pressure, which in some cases leads to achieve desired results at lower pressure [48,77].

The spores of pathogenic microorganisms in food are extremely pressure-resistant. Thus, pressure–temperature co-treatment is considered to be one of the most effective and feasible methods for the inactivation of different pathogenic spores [46,78,79]. Table 3 shows the inactivation effect of HPP and HPTP on certain microbial spores in different fruit and vegetable systems. Previous studies have also mentioned that pressure and moderate temperature exhibit a potent synergistic effect on the inactivation of microorganisms and enzymes [68]. Evelyn et al. [80] exposed apple juice inoculated with *Neosartorya fischeri* JCM 1740 spores to high-pressure (600 MPa, room temperature), and the control group was thermally treated at 75 °C. It was mentioned that HPP treatment (600 MPa, 75 °C, 10 min) was the best method for inactivation of *N*. *fischeri* JCM 1740 ascospores in apple juice with a 3.3 log cfu/mL reduction compared to no reduction followed by thermal treatment. Similar results were also reported by Filipa et al. [81], that no significant inactivation of *A. acidoterrestris* spores was observed in orange juice followed by temperature or pressure treatment alone.

However, HPP in conjunction with thermal treatment can effectively inactivate spores with a 2.7 log cfu/mL reduction at 600 MPa, 65 °C, and 10 min. Evelyn et al. [36] also reported that the combination of temperature and pressure treatment was more effective compared to either pressure or thermal alone for inactivating *Byssochlamys nivea* JCM 12,806 spores in strawberry puree. In addition, the combination of temperature and pressure was mentioned to be efficient compared to traditional thermal processing. As shown in Table 1, most of the models of high-pressure and moderate-temperature synergistic sterilization are nonlinear models. Hossein et al. [34] reported that Weibull distribution was the best mathematical model to describe the non-linear inactivation of *B. coagulans* 185A spores in tomato juice by high-pressure and moderate temperature synergistic process, whereas first-order kinetics were appropriate to explain microbial inactivation during thermal processing alone.

Evelyn et al. [36] simulated the effect of temperature on *Bys. nivea* JCM 12,806 ascospores in strawberry puree at 600 MPa. Weibull model efficiently described ascospore inactivation by HPP-thermal treatment (600 MPa and 38, 50, 60, 75 °C). In another study by Shao et al. [85], high-pressure treatment (350 MPa) at 10–30 °C for 5 min presented no significant impact on *Escherichia coli* K-12 count, whereas HPP at 40 °C was reported to reduce *E. coli* log survivors. This finding is attributed to the fact that temperature and pressure exhibit opposite effects on volume expansion. The increase in temperature contributes towards volume increase, while pressure has a reversed effect on volume. For similar reasons, some studies have also shown that for microorganisms at sublethal levels, the inactivation effect of pressure was elevated at lower temperatures [86]. In the following section, the effects of a combination of low temperature and high pressure on microorganisms have been reviewed in detail.

#### 3.3.2. High-Pressure and Low-Temperature Synergistic Sterilization in Fruits and Vegetables

**Figure 1:**
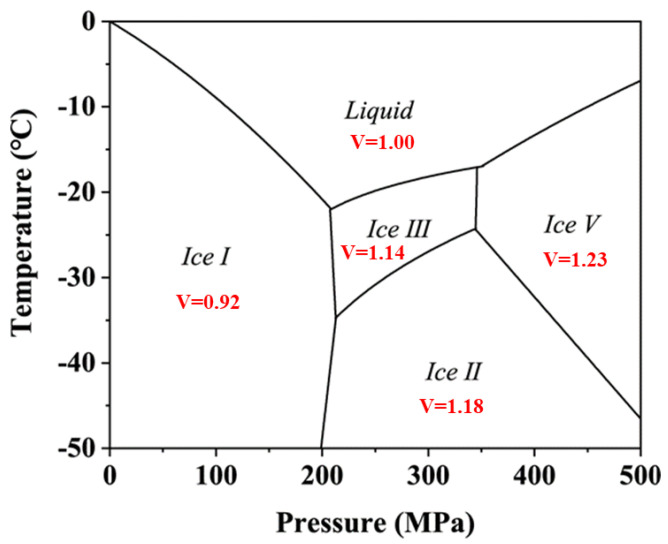
The volume of ice crystals (pure water) in individual phases; the letter V in the figure is the corresponding volume of different phases (g/cm^3^). The image was drawn concerning the data of Bridgman et al. [88] and Xiao et al. [89].

High-pressure freezing and thawing has gained the interest of food researchers due to its inherent property to retain the quality of food. High-pressure freezing can improve the freezing effect in essence by applying pressure during freezing at atmospheric pressure. By adjusting the phase transition temperature of the water, this method increases the degree of supercooling and freezing rate during the freezing process and then changes the path of crystal nucleus formation and ice crystal growth [87]. This method has the advantages of rapid heat and mass transfer, formation of small ice crystals during freezing, and even distribution of ice crystals in food tissues and low juice leakage rates during thawing. That in turn protects the texture and nutrient quality of food products [35]. Thus, high-pressure freezing and thawing resolve the problem of irreversible quality damage caused by traditional freeze–thaw processing and successfully meet consumers’ demand for high-quality frozen and thawed food. Unlike high-pressure and moderate-temperature synergistic sterilization, there is the problem of metastable phase transition of water (ice) during high-pressure and low-temperature synergistic sterilization. This metastable phase transition of water plays a crucial role in high-pressure and low-temperature synergistic sterilization. The metastable properties of ice crystals have often been neglected in previous studies on microbial decontamination involving ice I–ice III phase transitions. The temperature–pressure phase diagram of pure water is shown in Figure 1.

During the pressurization process, there are phase transitions from ice I to metastable ice I, metastable ice I to ice III, and recrystallization from water to ice III. However, there are phase transitions from ice III to ice I and from water to ice I during the pressure relief process. The transition from ice I to other ice phases results in an instantaneous decrease in system volume, as shown in Figure 1. This phase transition of ice crystals causes microbial cell damage by mechanical forces [90]. Pedro et al. and Luscher et al. have also mentioned that microbial inactivation during high-pressure and low-temperature synergistic sterilization is caused due to mechanical effects associated with phase transition from ice I to ice III [91,92]. Therefore, crystal formation and phase transition play an important role in the inactivation of pathogenic microorganisms in food samples during high-pressure and low-temperature synergistic treatment. It is important to note that the pressure tolerance of the majority of microbes is decreased at low temperatures. Thus, Su et al. [86] reported a combination or interaction effect of pressure and subzero temperature for the inactivation of microorganisms in food. It was mentioned that phase transitions of Ice I/Ice III by pressurizing frozen systems above 200 MPa are responsible for bacterial destruction [91]. Zhu et al. [35] showed that the high-pressure inactivation effect on *E. coli* in frozen carrot juice samples was better compared to microbial inactivation in unfrozen samples (Table 1). Researchers have reported a 1.87 log cfu/mL reduction for *E. coli* in unfrozen carrot juice, while a 6.80 log cfu/mL reduction was mentioned for *E. coli* in frozen carrot juice after treatment at 330 MPa for 10 min.

Moussa et al. [93] reported an approximate 4 log cfu/mL reduction in *E. coli* K-12TG1 count in Luria-Bertani medium (liquid, not frozen) followed by high-pressure treatment at 350 MPa for 10 min at −20 °C. However, in the case of frozen samples, a better bactericidal effect (5 log cfu/mL reduction) was observed after HPP at 330 MPa for 10 min. Wang et al. [84] reported 3.5 log cfu/mL reductions for *E. coli* ATCC 25,922 in frozen bayberry juice after treatment at 300 MPa for less than 5 s, but the same treatment only resulted in 1.2 log cfu/mL reductions in the case of unfrozen sample. In addition, an increase in the pressure holding time resulted in a potent antibacterial effect as *E. coli* was not detected in the samples after treatment at 300 MPa for 5 min at −5 °C. Sami et al. [83] also reported a 4.88 log cfu/mL reductions for *E. coli* in frozen orange juice (−80 °C) after treatment at 250 MPa for 15 min. However, without freezing, the same treatment resulted only in a 0.42 log cfu/mL reduction. Moussa et al. [93] reported that high-pressure treatment (150, 250 and 350 MPa) for 10 min at −20 and −10 °C presented a potent antibacterial effect against *E. coli* K-12TG1 compared to when the sample was treated at 25 °C. The above results show that the microbial inactivation by HPP treatment in frozen samples was higher compared to the unfrozen samples. In addition, below 0 °C, the inactivation effect of high-pressure treatment on tested pathogenic microorganisms was obvious.

In addition to the important role of crystal formation and phase transition in microbial inactivation in frozen samples during HPP, increased sensitivity of proteins to high pressure at low temperatures also leads to the rapid denaturation of proteins. That in turn plays a significant role in the reduction of the pressure resistance of microorganisms. Moreover, the structure of the cell membrane was more vulnerable to damage at low temperatures. In general, only a few studies have reported the impact of combined high-pressure and sub-zero temperatures on food sterilization. In particular, studies on the microbial inactivation kinetics of high-pressure sterilization at sub-zero (freezing) temperatures are scarce [92,94]. Thus, it is of immense importance to study food sterilization using high pressure in conjunction with low temperature (below zero) and its related kinetics.

### 3.4. Composition of Fruits and Vegetables and Food Additives

The biochemical composition of fruits and vegetables, such as protein, carbohydrate content, and soluble solid content, significantly influence the impact of HPP or HPTP on microbial inactivation [95]. Studies have shown that the biochemical components in the food matrix protect microbes and increase their resistance towards pressure [48,96,97]. In the fruit processing industry, soluble solids content was one of the most important parameters that affects microbial resistance towards pressure and heat [64,98,99]. Rafael et al. [100] reported that in the case of combined high-pressure and moderate temperature treatment, the low content of soluble solids content in the medium lead to higher inactivation of *A. acidoterrestris* spores. The *D*-values were 4.17, 7.59, and 13.71 min in broths having 10, 20, and 30 Brix, respectively, showing the protective effect of the soluble solid content against the HPP-thermal process. In general, due to the complex composition of food materials, it is necessary to use the actual food materials as a medium for inoculation during high-pressure treatment. In addition, to enhance the practical application of high-pressure processing, extensive research in this field still needs to be conducted.

Researchers are also using additives in the medium/food matrix to enhance the efficiency of HPP to inactivate pathogenic microorganisms or spores that were difficult to kill by HPP treatment alone. When an antibacterial agent was used as an additive, the bactericidal effect of high-pressure treatment will be enhanced significantly. Julie et al. [101] reported enhanced microbial inactivation by HPP treatment with the addition of natural antimicrobials. Chung et al. [102] studied the effect of high-pressure and the addition of tert-butylhydroquinone on *Listeria* spp. and *E. coli*, and the results showed that the addition of the additive significantly improve the bactericidal effect of high-pressure treatment. HPP combined with natural antimicrobial compounds can effectively eliminate the pressure-resistant subpopulation and inhibit their revival or resuscitation [103]. Pokhrel et al. [104] found that a more than 5 log reduction of both *L. innocua* and *E.coli* in carrot juice was achieved by the combination of HPP (300 MPa/35 °C/2 min) and natural bacteriocins, compared with less than 1 log reduction in the absence of natural bacteriocins. Zhao et al. found that HPP combined with natural bacteriocins had a significant synergistic effect on reducing the total aerobic bacteria in cucumber juice [105]. The advantage of high-pressure was that there is no specific requirement to add an artificial additive while maintaining better food quality. However, it is worth considering the addition of some natural antimicrobial compounds during the HPP to enhance its antimicrobial capability.

### 3.5. Water Activity and pH Value

Water availability also affects the resistance of microbial spores towards high-pressure or high-pressure and moderate-temperature synergistic treatment [106]. Thus, the water activity of food materials plays a very important role to change the resistance of microorganisms towards high-pressure treatment. Pressure transfer under high-pressure treatment depends on fluid, so it is not suitable to sterilize dried food, powder, or granular food. Decreased water activity causes cell shrinkage and cell membrane thickening, thereby reducing cell volume and cell membrane fluidity and permeability. Thus, when dealing with dry food material, the addition of water will significantly enhance the bactericidal effect of high-pressure treatment [96,107]. In addition, incomplete spore germination under low water availability conditions may also be one of the reasons for the change in the pressure resistance of microbes [108]. Rodriguez et al. [109] reported that the bactericidal effect of high-pressure on *E. coli* was significantly weakened when the water activity of samples was reduced. Moussa et al. also reported that low water activity significantly enhanced the pressure resistance of *S. cerevisiae* [110]. Due to the lack of regulations on the water activity of food material by food authorities, the water activity control due to changes in moisture content, temperature, humidity, and other environmental factors are difficult to change. Unil now, measures to control water activity have not been incorporated into high-pressure sterilization technology. Thus, extensive research is required to include water activity controllers in HPP technology to enhance its microbial inactivation capability.

The pH value of the fruits and vegetables also has a great influence on the pressure resistance of microorganisms during HPP or HPTP. Although pH alone may not be enough to inactivate microorganisms, its combination with high pressure greatly enhances treatment lethality. Microbes exposed to high-pressure processing experience irreversible damage, and cells that are not presenting any physical damage may become sensitive to the high acidity of the medium [48]. The pathogenic microorganisms and their spores present in acidic foods are less resistant to stress compared to the microbes present in low-acid or alkaline foods. In other words, harmful microorganisms and spores in high-acid or acidic foods can be killed with lower pressure and temperature than those in low-acid or alkaline foods. Previously, researchers have acidified foods with citric acid or ascorbic acid to improve the food safety and sterilization efficiency of high-pressure or temperature and combined treatment. High pressure is also reported to affect the pH of the food system, but not to a large extent. Zhang et al. [10] treated carambola puree with high pressure (0–800 MPa, at 25 °C) and found a slight decrease in the pH value of carambola puree with an increase in the pressure. The pH value of the control sample was 4.57, and after 800 MPa treatment, the pH value of the sample was 4.41. The compression of food might cause the ionization of molecules such as H_2_O, which increases the H^+^ ion concentration and affect the pH variation of HPP-treated food products [111,112].

Presently, in the bactericidal kinetics of high-pressure or combined temperature and pressure treatments, certain empirical models are extensively used to fit the bactericidal curve. However, various factors affecting the bactericidal effect such as pH, water activity, and natural antibacterial in the medium are not considered. Although curve fitting methods such as first-order kinetics and the Weibull model are effective in evaluating experimental data, other factors such as food composition, pH value, and water activity will also significantly affect the accuracy of the model prediction when establishing the kinetic model of high-pressure sterilization. Therefore, different models are required to predict the kinetics of HPP-induced microbial inactivation and to estimate the effect of HPP under different conditions. In addition, whatever the model is, it needs to be validated in other real fruit and vegetable systems before it can be routinely used throughout the fruit and vegetable processing industry.

## 4. Conclusions and Future Outlook

High-pressure induced microbial inactivation is also influenced by the temperature, pH, water activity, nutritional composition of fruits and vegetables, and type of microorganism and their growth phase. Therefore, other factors besides pressure should be considered in future inactivation modelling, especially treatment temperature, which can significantly affect inactivation mechanics. Among them, the bactericidal kinetics of low temperature combined with high pressure is the least explored area, and is worthy of further study owing to its inherent advantage of producing high-quality food products. On the other hand, most of the existing studies only use one or two models to simply fit the bactericidal kinetic curve; however, these studies lack optimization, verification, evaluation, and application of the bactericidal model. That in turn results in the poor credibility of the research conclusions and applicability of the model. In addition, in the bactericidal kinetics study of high-pressure or combined temperature and pressure treatment, some empirical models are mainly applied to fit the bactericidal curve, and various factors affecting the bactericidal effect are not considered comprehensively. Therefore, it is necessary to carry out extensive research to obtain more data and an efficient bactericidal kinetic model. In addition, inactivation models need to be validated in other food systems before they can be routinely used throughout the fruit and vegetable processing industry.

In addition, the high cost of high-pressure equipment and high initial investment cost is one of the important reasons that hinders the application of high-pressure technology in the food processing industry. At present, most high-pressure equipment used in the food industry has a rated pressure of ≤600 MPa. If the device operates at high pressure (>500 MPa) for a long time, the maintenance cost of the device will increase. Optimizing high-pressure production processes in food processing can save costs. Therefore, the research on high-pressure food processing technology is of great significance to the high-pressure food processing industry.

## Figures and Tables

**Table 1:** Application of HPP and HPTP bactericidal kinetic models in different fruit and vegetable systems.

Species	Food System	Pressure (MPa)	Temperature (°C)	Model Type	Model Parameters			Reference
					R^2^			
*E. coli*	Frozen carrot juice	200	−20	First-order	*D*-value (min)	4.03	0.963	[35]
		250				3.97	0.954	
		300				2.62	0.974	
		350				2.21	0.991	
		400				2.12	0.994	
	Unfrozen carrot juice	300	4	First-order		28.53	0.850	
		350				9.20	0.940	
		400				5.32	0.827	
*Bys. nivea* JCM 12,806 ascospores	Strawberry puree	600	50	Weibull	*b* = 0.16	*n* = 0.57	0.959	[36]
		600	60		*b* = 0.19	*n* = 0.65	0.993	
		600	75		*b* = 0.29	*n* = 0.66	0.997	
*B. coagulans* 185A spores	Tomato juice	0.1	100	First-order	*D*-value (min)	1.66	0.960	[34]
		0.1	105			0.59	0.970	
		600	75	Weibull	*b* = 0.87	*n* = 0.79	0.950	
		600	85		*b* = 1.28	*n* = 0.70	0.940	
		600	95		*b* = 1.93	*n* = 0.68	0.950	
*L. monocytogenes* 4a KUEN 136	Orange juice	300	25	First-order	*D*-value (min)	2.87	0.970	[37]
		400				1.80	0.980	
		600				0.87	0.940	
	Peach juice	300	25	First-order		6.17	0.950	
		400				3.39	0.960	
		600				1.52	0.970	

MPa, megapascal; *D*-value, decimal reduction time; *b* and *n* are the Weibull scale and shape factors (Equation (2)), respectively; R^2^, coefficient of determination.

**Table 2:** Inactivation effect of HPP on different kinds of microorganisms in the same food systems.

Species	Microbial Collection	Food System	Pressure (MPa)	Temperature (°C)	Holding Time (min)	Log Reduction	Reference
*Zygosaccharomyces bailii* ATCC 2333	American Type Culture Collection (ATCC) Manassas, VA, USA	Mango juice	300	20	5	2.7–2.9	[60]
*Pichia membranaefaciens* ATCC 2085						3.7–4.3	
*Leu. mesenteroides* ATCC 8293						<0.5	
*L. monocytogenes*	Food Microbiology Laboratory, New York State Agricultural Experiment Station (Geneva) and Food Safety Laboratory, Department of Food Science, Cornell University, Ithaca, NY, USA	Açaí juices	400	5	1	6–7	[61]
*E. coli* O157:H7						3–4	
*Salmonella* spp.						3–4	
*B. coagulans* 185A spores	Department of Animal & Food Sciences, University of Delaware, Newark, DE, USA	Tomato juice	600	95	1	3.3	[34]
*B. coagulans* 186A spores						3.9	
*B. coagulans* ATCC 7050 spores						4.5	

**Table 3:** Inactivation effect of HPP and HPTP on harmful microorganisms in different food systems.

Species	Microbial Collection	Food System	Pressure (MPa)	Holding Time (min)	Temperature (°C)	Log Reduction	Reference
*B. coagulans* ATCC 7050 spores	André Tosello Foundation, in Campinas, SP, Brazil	Tomato pulp	300	20	60	2.5	[82]
			500		60	4.5	
			600		60	6.5	
			300		50	<2	
			500		50	4.0	
			600		50	4.0	
*A. acidoterrestris* NZRM 4098 spores	New Zealand Reference Culture; Collection, Medical Section, Fort Richard Laboratories, New Zealand	Orange juice	600	10	45	<1	[81]
			600		55	1.2	
			600		65	2.7	
			200		65	1.7	
			0.1		65	-	
*N. fischeri* JCM 1740 ascospores	Japan Collection of Microorganisms, Tsukuba, Ibarak, Japan	Apple juice	0.1	30	75	-	[80]
			600		38	1.2	
			600		50	1.4	
			600		60	2.8	
			600		75	4.8	
*Bys. nivea* JCM 12,806 ascospores	Japan Collection of Microorganisms, Tsukuba, Ibarak, Japan	Strawberry puree	600	30	38	0.7	[36]
			600		50	1.1	
			600		60	1.8	
			600		70	2.8	
*E. coli* K12	Culture collection of the Department of Food and Nutritional Sciences, University of Reading, Reading, UK	Orange juice	250	15	4	0.42	[83]
			250		−80	4.88	
*E. coli* ATCC 25,922	-	Frozen bayberry juice	170	<5 s <5 s	−20	3	[84]
			250		−20	3.5	
			170	5	−20	ND	
		Unfrozen bayberry juice	170	<5 s <5 s	25	0.5	
			250		25	1.2	
			170	5	25	1.5	
*E. coli* ATCC 25,922	China General Microbiological Culture Collection Center, Beijing, China	Unfrozen carrot juice	300	10	4	<0.2	[35]
			400		4	1.7	
		Frozen carrot juice	300		−20	4	
			400		−20	5	

## Data Availability

Not applicable.
